# *In vivo* imaging of murid herpesvirus-4 infection

**DOI:** 10.1099/vir.0.006569-0

**Published:** 2009-01

**Authors:** Ricardo Milho, Christopher M. Smith, Sofia Marques, Marta Alenquer, Janet S. May, Laurent Gillet, Miguel Gaspar, Stacey Efstathiou, J. Pedro Simas, Philip G. Stevenson

**Affiliations:** 1 Division of Virology, Department of Pathology, University of Cambridge, UK; 2 Instituto de Microbiologia e Instituto de Medicina Molecular, Faculdade de Medicina, Universidade de Lisboa, Portugal

## Abstract

Luciferase-based imaging allows a global view of microbial pathogenesis. We applied this technique to gammaherpesvirus infection by inserting a luciferase expression cassette into the genome of murine herpesvirus-4 (MuHV-4). The recombinant virus strongly expressed luciferase in lytically infected cells without significant attenuation. We used it to compare different routes of virus inoculation. After intranasal infection of anaesthetized mice, luciferase was expressed in the nose and lungs for 7–10 days and in lymphoid tissue, most consistently the superficial cervical lymph nodes, for up to 30 days. Gastrointestinal infection was not observed. Intraperitoneal infection was very different to intranasal, with strong luciferase expression in the liver, kidneys, intestines, reproductive tract and spleen, but none in the nose or lungs. The nose has not previously been identified as a site of MuHV-4 infection. After intranasal infection of non-anaesthetized mice, it was the only site of non-lymphoid luciferase expression. Nevertheless, lymphoid colonization and persistence were still established, even at low inoculation doses. In contrast, virus delivered orally was very poorly infectious. Inoculation route therefore had a major impact on pathogenesis. Low dose intranasal infection without anaesthesia seems most likely to mimic natural transmission, and may therefore be particularly informative about normal viral gene functions.

## INTRODUCTION

Gammaherpesviruses are highly prevalent and cause considerable disease. A major challenge in combating this disease is to understand natural infection, for example how gammaherpesviruses first enter their hosts. Neither Epstein–Barr virus (EBV) nor Kaposi's sarcoma-associated herpesvirus (KSHV) are easy to analyse, because their infections are largely limited to humans. Gammaherpesviruses that allow experimental *in vivo* analysis can therefore tell us a great deal. Murine herpesvirus-4 (MuHV-4) (Nash *et al.*, 2001; Stevenson & Efstathiou, 2005) currently provides the most accessible model. The archetypal MHV-68 strain came from a bank vole (*Myodes glareolus*) (Blaskovic *et al.*, 1980). However, MuHV-4 has also been isolated from yellow-necked mice (*Apodemus flavicollis*) (Kozuch *et al.*, 1993), and closely related viruses have been isolated from a shrew (*Crocidura russula*) (Chastel *et al.*, 1994) and from wood mice (*Apodemus sylvaticus*) (Blasdell *et al.*, 2003), suggesting that MuHV-4-like viruses have wide host ranges. Although MuHV-4 has not been isolated from house mice (*Mus musculus*), the natural correlate of inbred laboratory strains, its benign persistence in laboratory mice contrasts with the high pathogenicity or marked attenuation generally shown by herpesviruses in xenogenic hosts (Stevenson *et al.*, 2002a). There is serological evidence that MuHV-4 (or a close relative) naturally infects house mice (Mistrikova *et al.*, 2000), and MuHV-4 major histocompatibility complex (MHC) class I degradation (Boname & Stevenson, 2001; Lybarger *et al.*, 2003), transporter associated with antigen processing (TAP) degradation (Boname *et al.*, 2004), complement inhibition (Kapadia *et al.*, 1999) and chemokine binding (Parry *et al.*, 2000; van Berkel *et al.*, 2000) all work in laboratory mice. Such immune evasion functions are typically species-specific, for example MuHV-4 K3 downregulates MHC class I expression poorly in rat cells (our unpublished data). MuHV-4 infection of laboratory mice therefore seems to provide a reasonable pathogenesis model.

Experimental MuHV-4 infection typically employs intranasal virus inoculation under general anaesthesia. This leads to a lytic infection of lung alveolar epithelial cells that is controlled within 2 weeks (Nash & Sunil-Chandra, 1994). Virus meanwhile seeds to lymphoid tissue and drives the proliferation of latently infected B cells. This peaks at 2 weeks post-infection (p.i.) and is controlled by 4 weeks. A predominantly latent infection of memory B cells (Flano *et al.*, 2002) then persists. In addition to intranasal infection, MuHV-4 has been given intraperitoneally (Weck *et al.*, 1996), subcutaneously (Raslova *et al.*, 2001), intravenously (Sunil-Chandra *et al.*, 1992), orally (Blaskovic *et al.*, 1984), intracerebrally (Terry *et al.*, 2000) and by gavage (Peacock & Bost, 2000). Its capacity to infect many different anatomical sites corresponds to a broad tropism for different fibroblast and epithelial cell lines (Gillet *et al.*, 2007a).

All inoculation routes lead to B-cell infection, and latently infected B cells can in theory transport MuHV-4 from any one site to any other. It is therefore often assumed that the different modes of infection are fairly equivalent. However, the peak of B-cell colonization coincides with a strong CD8^+^ T-cell response (Stevenson *et al.*, 1999a) that limits lytic spread (Stevenson *et al.*, 1999b), and there is evidence from EBV that latently infected B cells recirculate mainly through their site of initial infection (Laichalk *et al.*, 2002). The exposure of different epithelial and fibroblast populations to MuHV-4 infection may therefore depend strongly on inoculation route. Even without a direct comparison, it is clear that inoculation route can affect experimental outcomes. For example, intraperitoneal MuHV-4 infection led to the conclusion that B cells support acute lytic replication and macrophages support long-term latency (Weck *et al.*, 1996), whereas after intranasal infection, macrophage colonization is transient and B cells provide the long-term latent reservoir (Sunil-Chandra *et al.*, 1992).

A major impetus to establishing a realistic form of experimental MuHV-4 infection has been the finding that *in vitro* and *in vivo* virus neutralization are quite different (Gillet et al., 2007b). The implication is that to understand neutralization, we must also understand host entry. Global imaging provides one way to compare different infection routes. Here we imaged MuHV-4 lytic gene expression by luciferase expression and charge-coupled-device camera scanning (Hutchens & Luker, 2007). After intranasal virus inoculation without anaesthesia, luciferase expression was limited to the nose and superficial cervical lymph nodes (SCLN), but still established a persistent infection. In contrast, orally delivered virus was poorly infectious. The nose therefore seems a likely physiological route of host entry. Studies of infection by this route may give new insights into MuHV-4 gene functions.

## METHODS

### Mice.

Female BALB/c mice were infected with MuHV-4 when 6–12 weeks old. Intranasal infections with anaesthesia were in 30 μl aliquots, those without were in 5 μl. All experiments conformed to local animal ethics regulations; those in Cambridge also followed Home Office Project Licence 80/1992. For luciferase imaging, mice were injected intraperitoneally with luciferin, anaesthetized with ketamine/xylazine or isoflurane, then scanned with an IVIS Lumina (Caliper Life Sciences). In preliminary experiments we used the manufacturer's recommended dose of 150 μg luciferin g^−1^. In later experiments this was reduced to 2 mg/mouse without noticeably less signal. Signal intensity was fairly constant between 3 and 10 min after injection. Mice were routinely imaged after 5 min. For quantitative comparisons, we used Living Image software (Caliper Life Sciences) to obtain the maximum radiance (photons per s per cm^2^ per steradian, i.e. photons s^−1^ cm^−2^ sr^−1^) over each region of interest, relative to a negative control region.

### Cells.

Baby hamster kidney (BHK-21) cells, NIH-3T3 cells, NIH-3T3-CRE cells (Stevenson *et al.*, 2002b) and NIH-3T3-TET50 cells were propagated in Dulbecco's modified Eagle's medium (Invitrogen) supplemented with 2 mM glutamine, 100 U penicillin ml^−1^, 100 μg streptomycin ml^−1^ and 10 % fetal calf serum. NIH-3T3-TET50 cells were made by serially transducing NIH-3T3 cells with three retroviruses: one expressed ORF50 from a promoter with doxycycline-responsive (TRE) promoter; one expressed constitutively a transcriptional suppressor with doxycycline-inactivated TRE-binding; and one expressed constitutively a transcriptional activator with doxycycline-activated TRE-binding. All together allowed doxycycline-inducible ORF50 expression. The ORF50 coding sequence was amplified by PCR from infected cell cDNA and cloned into pREV-TRE (Clontech). The TRE-binding transcriptional suppressor was excised from pTET-tTS (Clontech) with *Eco*RI/*Cla*I, the *Cla*I site was blunted with T4 DNA polymerase and the fragment was ligated into the *Eco*RI-blunted *Xho*I sites of pMSCV-IRES-PURO. The TRE-binding transcriptional activator was from pREV-TET-ON (Clontech). Each plasmid was transfected into 293T cells together with the pEQPAM3 packaging plasmid (Persons *et al.*, 1998). Retroviruses were collected after 48 and 72 h, and added to cells with 6 μg polybrene ml^−1^. Triply transduced cells were selected with puromycin+hygromycin+G418.

### Viruses.

The luciferase coding sequence plus polyadenylation signal was removed from pGL4.10 (Promega) by digestion with *Bgl*II/*Sal*I and cloned into the *Bam*HI/*Sal*I sites of pSP73, downstream of a 500 bp MuHV-4 M3 promoter (May *et al.*, 2005a). M3-luciferase-polyA was then excised with *Bgl*II/*Sal*I, blunted with Klenow fragment DNA polymerase and cloned into the blunted *Mfe*I site (genomic co-ordinate 77 176, GenBank accession no. U97553) of a *Bgl*II MuHV-4 genomic clone (co-ordinates 75 338–78 717), again in pSP73. The expression cassette plus genomic flanks was subcloned into the *Bam*HI site of the pST76K-SR shuttle vector and recombined into a MuHV-4 bacterial artificial chromosome (BAC; Adler *et al.*, 2000). An ORF50-deficient derivative was made by cloning a *Hin*cII genomic fragment (co-ordinates 63 844–70 433) into the *Hin*cII site of pUC9 (New England Biolabs), with the *Bam*HI site of pUC9 at the 70 433 end of the insert. This was cut with *Bsm*I (67 792) and *Cla*I (69 177) to remove most of ORF50 exon 2 (67 661–69 376), blunted and dephosphorylated with Antarctic alkaline phosphatase (New England Biolabs). The eGFP coding sequence from pEGFP-N3 (Clontech) was ligated in place of the removed fragment. EGFP plus its genomic flanks was then excised using a genomic *Kpn*I site (66 120) and the *Bam*HI site in pUC9, cloned into the *Bam*HI/*Kpn*I sites of pST76K-SR, and recombined into the M3-LUC BAC. M3-LUC virus was recovered by transfecting BAC DNA into BHK-21 cells. For *in vivo* experiments, its *loxP*-flanked BAC/eGFP cassette was removed by passage through NIH-3T3-CRE cells. Virus stocks were grown in BHK-21 cells (Coleman *et al.*, 2003). ORF50^−^M3-LUC virus was recovered by transfecting BAC DNA into NIH-3T3-TET50 cells and propagated by treating the cells with doxycycline.

### Viral infectivity assays.

Virus stocks were titrated by plaque assay on BHK-21 cells (de Lima *et al.*, 2004), or on doxycycline-treated NIH-3T3-TET50 cells for ORF50^−^M3-LUC. Cell monolayers were incubated with virus (2 h, 37 °C), overlaid with 0.3 % carboxymethylcellulose, and 4 days later fixed and stained for plaque counting. Infectious virus in lungs was measured by freeze–thawing the lungs and homogenizing them in 1 ml complete medium prior to plaque assay. Latent virus was measured by infectious centre assay (de Lima *et al.*, 2004): spleen cells were co-cultured with BHK-21 cells, then fixed and stained for plaque counting after 4 days. Plaque assay titres of freeze–thawed lymphoid homogenates were always <1 % of infectious centre assay titres, so the latter essentially measured reactivable latent virus.

### Viral genome quantification.

Viral genome loads were measured by real-time PCR (Gaspar *et al.*, 2008). DNA from organs (50–80 ng) was used to amplify MuHV-4 genomic co-ordinates 4166–4252 (Rotor Gene 3000; Corbett Research). The PCR products were quantified by hybridization with a TaqMan probe (genomic co-ordinates 4218–4189) and converted to genome copies by comparison with a standard curve of cloned plasmid template amplified in parallel. Cellular DNA was quantified in parallel by amplifying part of the adenosine phosphoribosyl transferase gene (forward primer 5′-GGGGCAAAACCAAAAAAGGA-3′, reverse primer 5′-TGTGTGTGGGGCCTGAGTC-3′, probe 5′-TGCCTAAACACAAGCATCCCTACCTCAA-3′).

### Southern blotting.

Viral DNA was extracted by alkaline lysis (Coleman *et al.*, 2003), digested, electrophoresed and transferred to nylon membranes (Roche Diagnostics). A [^32^P]dCTP-labelled probe (APBiotech) was generated by random primer extension (DECAprime II kit; Ambion). Membranes were hybridized with probe (65 °C, 18 h), washed in 30 mM sodium chloride, 3 mM sodium citrate, 0.1 % SDS solution at 65 °C and exposed to X-ray film.

### *In vitro* luciferase assays.

Cells were washed twice in PBS, then lysed in 1 % Triton X-100 (15 min, 4 °C). Cell debris was pelleted (13 000 ***g***, 5 min) and supernatants incubated in 20 mM glycylglycine, 20 mM potassium phosphate buffer (pH 7.8) with 1 mM dithiothreitol, 10 mM MgSO_4_, 3 mM EGTA, 2.5 mM ATP and 100 μM luciferin. The light emission of triplicate samples was detected by luminometry (Hewlett Packard).

### ELISA.

MuHV-4 virions were recovered from infected cell supernatants by ultracentrifugation, disrupted with 0.05 % Triton X-100 in 50 mM sodium carbonate buffer (pH 8.5), and coated onto MaxiSorp ELISA plates (Nunc). The plates were washed three times in PBS containing 0.1 % Tween 20, blocked with 2 % BSA in PBS 0.1 % Tween 20, then incubated with threefold serum dilutions from MuHV-4-exposed mice (1 h, 23 °C). The plates were then washed four times in PBS 0.1 % Tween 20, incubated (1 h, 23 °C) with alkaline phosphatase-conjugated goat anti-mouse IgG-Fc pAb (Sigma), washed five times, and developed with nitrophenylphosphate (Sigma). Absorbance was measured at 405 nm (Bio-Rad).

## RESULTS

### Generation and *in vitro* analysis of MuHV-4 expressing luciferase

An ideal luciferase reporter would mark both lytically and latently infected cells. However, high level latent gene expression is probably incompatible with normal host colonization for a gammaherpesvirus: most EBV-infected cells express no latency genes at all (Thorley-Lawson, 2001); both EBV and MuHV-4 limit antigen presentation from their episome maintenance proteins by low turnover (Yin *et al.*, 2003; Bennett *et al.*, 2005); and bypassing this evasion severely attenuates MuHV-4 latency (Bennett *et al.*, 2005). Autonomous promoters such as that of the human cytomegalovirus (HCMV) IE1 gene (Rosa *et al.*, 2007; Smith *et al.*, 2007) may therefore expose latently infected cells to unphysiological CD8^+^ T-cell recognition. This would explain why HCMV IE1-driven reporter gene expression is associated with marked *in vivo* MuHV-4 attenuation (Adler *et al.*, 2001).

We therefore aimed for lytic reporter gene expression, using as a promoter an ectopic copy of the 500 bp upstream of the MuHV-4 M3, an abundant early/late lytic gene (van Berkel *et al.*, 1999). *In situ* detection of M3 mRNA in infected lymphoid tissue (Simas *et al.*, 1999) and a latency establishment deficit of MuHV-4 M3 mutants (Bridgeman *et al.*, 2001) suggest that M3 might also be transcribed in latency. However, other early lytic transcripts are detectable in spleens (Marques *et al.*, 2003) – B cells even drive a substantial lytic antigen-specific CD8^+^ T-cell response (Stevenson *et al.*, 1999a) – and the latency deficit of M3 mutants may simply reflect that lytically infected cells no longer secrete M3 to provide bystander protection for their latently infected neighbours (Rice *et al.*, 2002; Stevenson, 2004).

**Fig. 1. f1:**
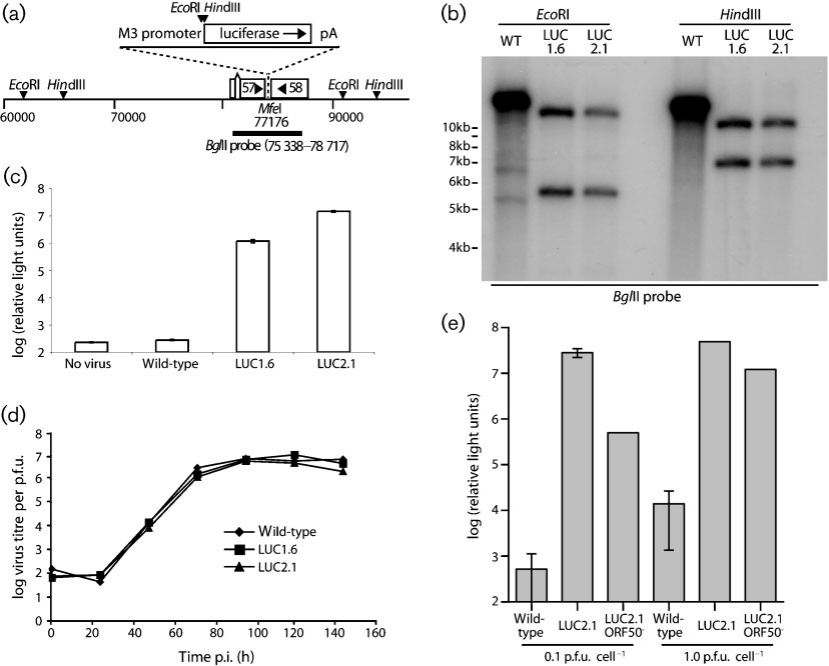
Generation of MuHV-4 expressing luciferase. (a) A 2 kb luciferase-polyA cassette was placed downstream of a 500 bp M3 promoter, in a *Mfe*I restriction site between ORFs 57 and 58. Relevant restrictions sites are shown. (b) Viral DNA was digested with *Eco*RI or *Hin*dIII and probed with the 75 338–78 717 *Bgl*II clone shown in (a). The luciferase expression cassette changes a 14.9 kb *Eco*RI band to 5.5 kb+12.0 kb, and a 14.5 kb *Hin*dIII band to 6.8 kb+10.1 kb. M3-LUC1.6 and M3-LUC2.1 are independently generated recombinant viruses. (c) BHK-21 cells were left uninfected or infected overnight (1 p.f.u. cell^−1^), then lysed and assayed for luciferase expression. Each bar shows the mean±sd of triplicate cultures. (d) BHK-21 cells were infected with wild-type or M3-LUC MuHV-4 (0.01 p.f.u. cell^−1^, 2 h), washed with PBS to remove unbound virions, then incubated at 37 °C. The infectious virus in each culture was measured by plaque assay. (e) BHK-21 cells were infected with wild-type MuHV-4, the M3-LUC2.1 recombinant or its ORF50^−^ derivative. Luciferase expression was assayed 18 h later by luminometry. Each bar shows mean±sd of five replicate infections. The ORF50^−^ cultures contained no replication-competent virus by plaque assay.

The luciferase expression cassette was inserted between the polyadenylation signals of ORFs 57 and 58 (Fig. 1a[Fig f1]). Southern blotting (Fig. 1b[Fig f1]) confirmed the predicted structures of recombinant viral genomes. Infected cells showed strong luciferase expression (Fig. 1c[Fig f1]). M3-LUC viruses showed no *in vitro* lytic replication defect (Fig. 1d[Fig f1]). Since ORF57 is essential for lytic replication (Song *et al.*, 2005) and ORF58 contributes to inter-cellular viral spread (May *et al.*, 2005b), luciferase expression appeared not to compromise the functions of neighbouring genes. Gp48 expression, which is ORF58-dependent, was also normal (data not shown).

We tested whether M3-LUC luciferase expression depended on the ORF50 lytic transactivator by generating an ORF50-deficient derivative (Fig. 1e[Fig f1]). This was propagated in complementing NIH-3T3-TRE50 cells and then tested for luciferase expression in non-complementing BHK-21 cells. At 0.1 p.f.u. cell^−1^, ORF50 disruption reduced the luciferase signal by 98–99 %. At 1 p.f.u. cell^−1^ the reduction was 80 %. Inhibiting viral DNA replication with phosphonoacetic acid had no effect on luciferase expression (data not shown). Thus, luciferase expression corresponded mainly to early lytic gene expression, with some additional ORF50-independent expression at high multiplicity infection. *In vivo* cells may differ from transformed cell lines in viral transcription, but it seemed unlikely that luciferase expression would reveal latent infection. We considered it to mark early lytic gene expression.

### *In vivo* analysis of MuHV-4 expressing luciferase

**Fig. 2. f2:**
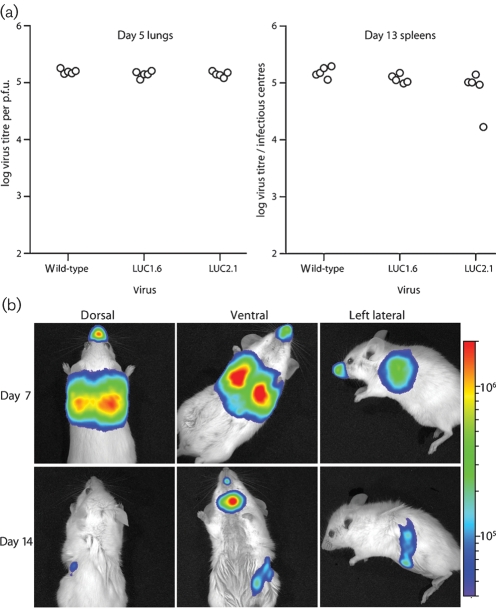
*In vivo* infection by luciferase-expressing MuHV-4. (a) M3-LUC1.6 and M3-LUC2.1 were compared with wild-type MuHV-4 for their capacity to colonize mice after intranasal infection. Lytic replication was tested by plaque assay of lungs after 5 days. Latency establishment was tested by infectious centre assay of spleens after 13 days. Each point shows the titre of one mouse. There was no significant difference between each virus. (b) Mice were infected intranasally (10^4^ p.f.u.) with M3-LUC2.1 MuHV-4 under general anaesthesia, and then injected with luciferin and imaged every 3–4 days. Images show a representative mouse at day 7 and 14 p.i. The signal in the mouth was atypical and probably corresponds to the strong neck signal reflecting off the incisors. The scale bar shows photons s^−1^ cm^−2^ sr^−1^.

We have previously shown that M3 promoter-driven ovalbumin or eGFP expression has a minimal effect on MuHV-4 replication *in vivo* (Smith *et al.*, 2006). M3-LUC viruses similarly showed normal replication after intranasal inoculation (Fig. 2a[Fig f2]). We selected M3-LUC2.1 for further analysis. We infected anaesthetized mice intranasally, then monitored luciferase expression by luciferin injection and charge-coupled-device camera scanning (Fig. 2b[Fig f2]). In preliminary experiments, removing the fur from mice prior to imaging had little effect on the signal obtained. Therefore in subsequent experiments no fur was removed. At the peak of lytic replication (5–7 days p.i.), a strong signal was visible in the thorax and the nose. At the peak of latency amplification (13–14 days p.i.), luciferase signals were weak or undetectable in the thorax and nose, but strong in the neck. Some mice also showed a weaker abdominal signal.

**Fig. 3. f3:**
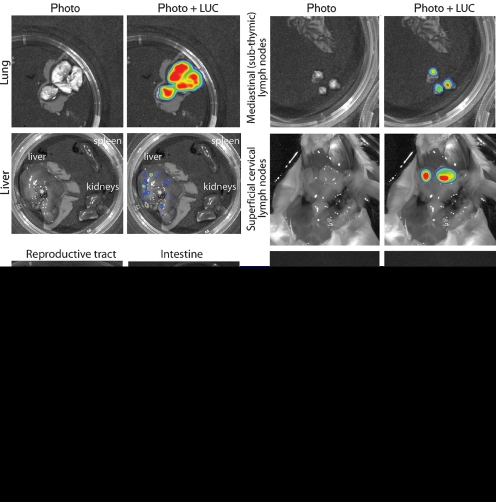
Luciferase signals from isolated organs after intranasal MuHV-4 infection. Mice equivalent to those in Fig. 2(b) were dissected and their organs imaged *ex vivo*. Each image is representative of data from at least five mice, and shows either a standard photograph (Photo) or that photograph overlaid with the luciferase signal (Photo+LUC). The colour scheme for relative signal intensity is as for Fig. 2.

*Ex vivo* imaging of dissected organs (Fig. 3) established the sources of the live images. The thoracic signal came from the lungs; the abdominal signal came from the spleen; and the neck signal came from the SCLN, particularly the most rostral node that lies alongside the salivary glands. The mediastinal (subthymic) and deep cervical LN showed weaker signals. Despite strong signals from nose and lungs, we saw no signal from the intervening trachea or main bronchi. Although MuHV-4 has been reported to infect mice after inoculation into the stomach (Peacock & Bost, 2000), and at least some of a 30 μl intranasal inoculum is likely to be swallowed, we saw no sign of intestinal infection. A signal was occasionally observed (10–20 % of mice) in the liver at day 7 (Fig. 3) and in abdominal LN at day 10–14 (data not shown). This could have reflected some intestinal infection. But it could also have reflected contiguous spread from the lungs and diaphragm or systemic virus spread via B cells. Other abdominal organs, such as the kidneys and reproductive tract, were consistently negative.

### Quantification of luciferase signals

**Fig. 4. f4:**
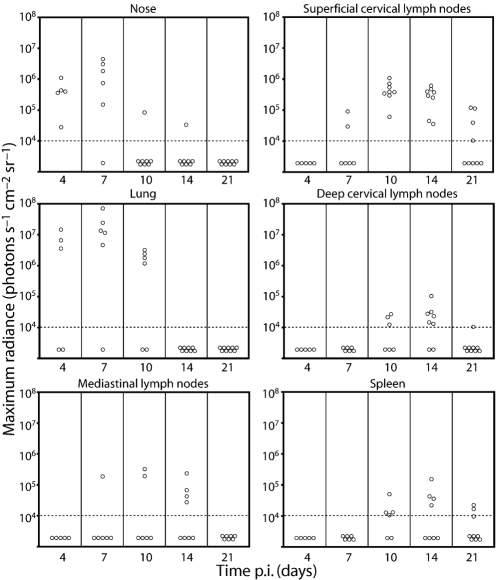
Quantification of luciferase signals from isolated organs after intranasal MuHV-4 infection. Mice were infected intranasally (10^4^ p.f.u.) with M3-LUC. At each time point, at least five mice per group were dissected for *ex vivo* organ imaging. Each point shows the maximum radiance value for one mouse. The horizontal dashed lines mark an arbitrary sensitivity threshold, chosen to minimize the chance of artefactual signals such as secondary light reflections.

Luciferase signals were quantified as the maximum radiance (photons s^−1^ cm^−2^ sr^−1^) over a region of interest – typically one dissected organ. Fig. 4 shows a time-course after intranasal infection. Occasional mice showed no signal even at peak average time points. Tracking individual mice showed that none remained uninfected. Rather the kinetics of luciferase expression varied; total signals over the whole time-course were similar. This possibly reflected variation in inoculum dose to the lung: some virus may have been trapped in nasal sinuses or coughed up after recovery from anaesthesia. SCLN signals were the most long-lived, sometimes persisting even at day 25 p.i. But generally all sites were negative by day 30.

Several caveats apply to live imaging. Overlying tissues clearly reduced light transmission, as neck signals were evident ventrally but not dorsally, and the dissected organs signals were stronger than live images. This limits the interpretation of fine quantitative differences. Mediastinal LN and deep cervical LN signals were only evident after dissection because they were obscured by those of the lungs and SCLN. Very strong signals sometimes gave secondary reflections. For example, in the day 14 ventral view (Fig. 2b[Fig f2]), the neck signal is reflecting off the incisors: after dissection, no mouth signal was observed. However, there was generally a good correlation between live images and dissected organ signals.

Since luciferase expression corresponded to early lytic gene expression, a lack of signal did not necessarily imply a lack of infection: tightly maintained viral latency might be missed. The lytic antigen-specific CD8^+^ T-cell responses generated by 7–10 days p.i. (Stevenson & Doherty, 1998) would also be expected to suppress luciferase expression. Nevertheless, luciferase signals appeared to match quite well the results of other assays (Sunil-Chandra *et al.*, 1992): an acute lytic infection in the lung (day 4–10) progressed to a subacute infection of lymphoid tissue (day 10–25), and both were largely resolved by day 30. Although MuHV-4 does not show significant productive infection in lymphoid tissue after intranasal inoculation (Nash & Sunil-Chandra, 1994), luciferase signals here were entirely consistent with evidence of early lytic gene expression (Stevenson *et al.*, 1999a; Liu *et al.*, 1999; Marques *et al.*, 2003). Thus, early lytic luciferase expression revealed both productive lytic replication and acute lymphoid colonization.

### Intraperitoneal infection

**Fig. 5. f5:**
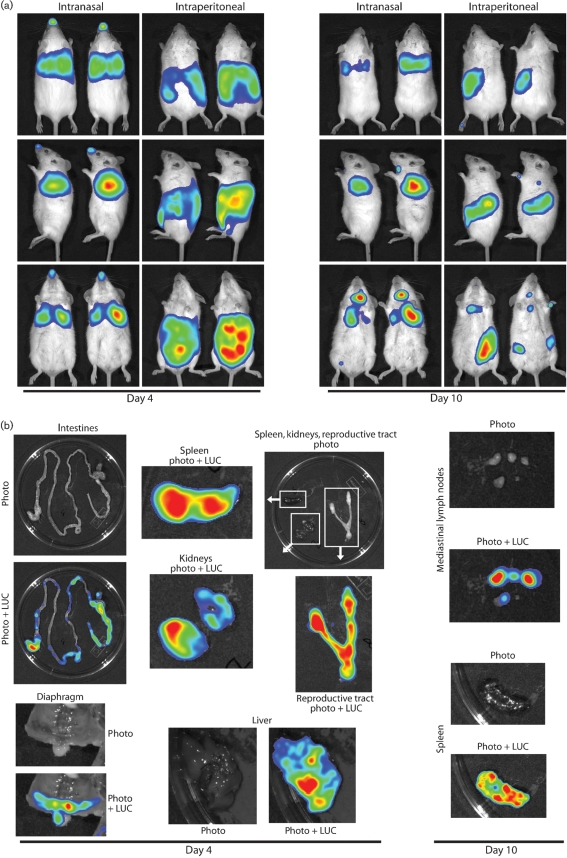
Comparison of intranasal and intraperitoneal MuHV-4 infections. (a) Mice were inoculated intranasally or intraperitoneally with 10^4^ p.f.u. of M3-LUC, then monitored for luciferase expression. Representative pairs of mice are shown. (b) Mice were dissected at 4 or 10 days after intraperitoneal virus inoculation to identify the source of the live imaging signals. The colour scheme for relative signal intensity is as for Fig. 2.

Many MuHV-4 pathogenesis studies have used intraperitoneal infection rather than intranasal (Speck & Virgin, 1999). Understanding the relationship between these infections is therefore important for integrating existing pathogenesis data into a coherent whole. Luciferase expression patterns after intraperitoneal or intranasal M3-LUC inoculations were markedly different (Fig. 5a[Fig f5]). At day 4 after intraperitoneal inoculation, there was a strong signal from the abdominal cavity and none from the nose or lungs. At the same time after intranasal inoculation, there was a strong signal from the lungs and none from the abdomen. By 10 days after intraperitoneal inoculation, there was still no signal from the lungs. The abdominal signal had decreased, and the only spread was to the mediastinal LN, which receive lymphatic drainage from the peritoneal cavity; the strong SCLN signal associated with intranasal infection remained weak or absent.

*Ex vivo* imaging (Fig. 5b[Fig f5]) showed that the abdominal signal of intraperitoneal infection had many sources, including the liver, spleen, kidneys and reproductive tract. The gut was also patchily positive, presumably reflecting serosal infection. Therefore, although MuHV-4 reached lymphoid tissue by either route, the distributions of lytic infection were almost mutually exclusive; in neither case did infected B cells appear to seed major new non-lymphoid sites of lytic gene expression. Even the spleen signals differed: intraperitoneal infection showed strong luciferase expression early on, consistent with productive infection (Weck *et al.*, 1996), while intranasal infection showed later, weaker expression, consistent with a predominantly latent infection. These data explained why intraperitoneal and intranasal infections have given such different phenotypes, for example with M11 knockouts (Gangappa *et al.*, 2002; de Lima *et al.*, 2005).

### Different infection doses

**Fig. 6. f6:**
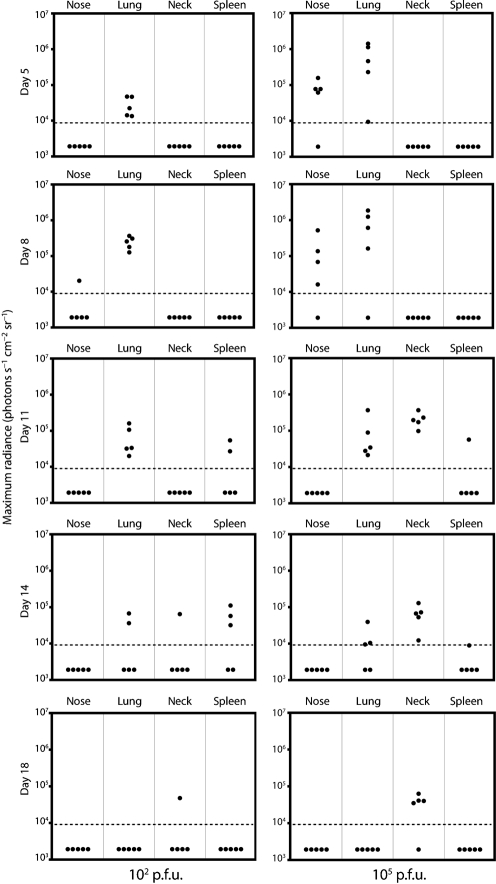
High dose and low dose intranasal infections. Mice were infected intranasally with M3-LUC (10^2^ or 10^5^ p.f.u.) under general anaesthesia, then live-imaged for luciferase expression every 3–5 days. Each point corresponds to one mouse. The horizontal dashed lines mark an arbitrary sensitivity threshold.

We next tested different inoculation doses via the intranasal route with general anaesthesia (Fig. 6). High dose infection (10^5^ p.f.u.) gave a luciferase signal in the lung that peaked at 4–7 days. After low dose infection (10^2^ p.f.u.) the signal peaked at 8–11 days and was generally less extensive. Both infections led to lymphoid colonization. Thus, beyond more virus giving more extensive early lytic replication, there was little difference between low and high doses, consistent with infectivity assays (Tibbetts *et al.*, 2003). Luciferase signals in the nose were uncommon after low dose infection, presumably because all the infectious particles ended up in the lungs. SCLN signals were also weak, implying that this site is colonized principally via the upper respiratory tract.

### Intranasal infection without anaesthesia

**Fig. 7. f7:**
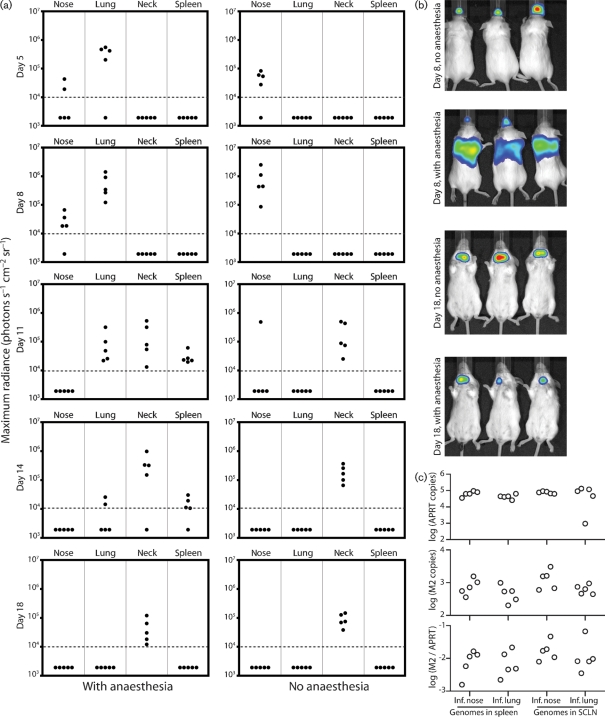
Intranasal infection with and without anaesthesia. (a) Mice were infected intranasally with M3-LUC (10^4^ p.f.u.), either awake or while anaesthetized, then imaged for luciferase expression every 3–5 days. Each point corresponds to one mouse. The horizontal dashed lines mark an arbitrary sensitivity threshold. (b) Representative images from the data summarized in (a). (c) At 1 month p.i., mice were analysed for viral genome loads in the spleen and SCLN by real-time PCR. The top panel shows the cellular control (APRT), the middle panel the MuHV-4 M2 gene, and the bottom panel the M2 load normalized by APRT. Each point corresponds to one mouse. There was no statistically significant difference between mice given anaesthesia (Inf. lung) or not (Inf. nose).

The prominent luciferase signals in noses after intranasal inoculation suggested that host entry might occur via the upper respiratory tract as well as via the lungs, where it has mostly been studied to date. We tested this by intranasal infection without anaesthesia (Fig. 7). As before, the anaesthetized controls showed strong luciferase signals in lungs and less consistent signals in the nose (Fig. 7a, b[Fig f7]). Infection then spread to the SCLN and spleen. Non-anaesthetized mice given the same virus dose showed a strong signal in the nose and none in the lungs. Thus, infection did not reach the lungs without anaesthesia. Non-anaesthetized mice also showed no luciferase signal in the spleen. However, their SCLN signals were at least as strong as those of anaesthetized mice. The lack of spleen signal presumably reflected that it was colonized relatively late in infection, when lytic antigen-specific CD8^+^ T-cell responses were strong. Real-time PCR quantification of viral genomes (Fig. 7c[Fig f7]) established that virus delivered to the nose still established normal persistence in both the SCLN and spleen.

### Oral infection

**Fig. 8. f8:**
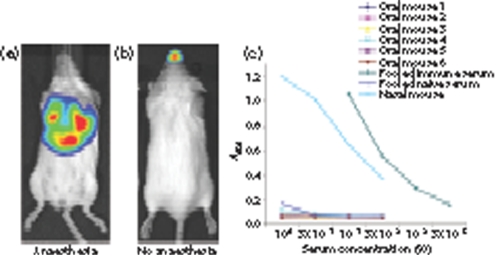
Oral infection. (a) Representative image of a luciferase-positive mouse 8 days after oral infection under general anaesthesia. (b) Representative image of a luciferase-positive mouse 7 days after oral infection without anaesthesia. (c) Sera from mice 1 month after oral inoculation without anaesthesia (50 p.f.u.) were analysed for MuHV-4-specific IgG by ELISA, along with pooled naive mouse serum, pooled immune serum and serum from a mouse infected (10 p.f.u.) intranasally without anaesthesia.

We also tested oral infection (Fig. 8). A 10^4^ p.f.u. inoculum with general anaesthesia gave no luciferase signal in 3/5 mice. What mice were infected showed the typical lung-dominated pattern of intranasal infection (Fig. 8a[Fig f8]), suggesting aspiration of the oral inoculum. A 10^6^ p.f.u. inoculum without anaesthesia gave luciferase signals in 5/8 mice. This time, the infected mice had the typical pattern of nose infection (Fig. 8b[Fig f8]), suggesting that here some of the oral inoculum reached the nose. Thus, there was no evidence of host entry by the oral route: normal infection was established if virions reached the nose or lung, but there was no infection if they did not.

Lower dose infections without anaesthesia – reducing the chance of inadvertent respiratory tract contamination – gave no luciferase signal (0/10 mice for 10^3^ p.f.u.; 0/24 mice for 50 p.f.u.), even when we imaged dissected organs. In contrast, a 10 p.f.u. intranasal inoculum without anaesthesia gave strong luciferase signals in 12/12 mice. A lack of infection by 50 p.f.u. of oral virus was confirmed by ELISA for virus-specific serum IgG at 1 month post-exposure (Fig. 8c[Fig f8]). PCR of spleen cell DNA for viral genomes at 15 days after oral inoculation was also completely negative in 6/6 mice (<5 viral genome copies per 80 ng DNA). Thus in adult mice, oral MuHV-4 was poorly infectious.

## DISCUSSION

How do herpesviruses enter their hosts? We compared different modes of experimental MuHV-4 infection, aiming to establish a plausible correlate of natural transmission for adult mice. For an anatomically complete view, we monitored infection by luciferase expression from the viral genome. Analysis of the standard intranasal infection model validated this approach. Thus, strong luciferase expression in the lung, weaker expression in lymphoid tissue, then quiescence, matched the consensus picture from other assays of productive viral replication in the lung, latency amplification with some lytic gene expression in lymphoid tissue, then immune control. Different modes of virus inoculation gave markedly different patterns of luciferase expression. Intranasal inoculation under general anaesthesia gave strong expression in the nose and lungs and weaker expression in lymphoid tissue; intraperitoneal inoculation gave strong expression in multiple abdominal organs, including the spleen, and none in the respiratory tract; intranasal inoculation without anaesthesia gave expression in just the nose and draining lymphoid tissue; and oral inoculation gave no expression at all. Normal transmission is presumably mucosal. The nose – but not the lung – is therefore a likely point of normal host entry.

The poor infectivity of oral MuHV-4 was surprising, as human gammaherpesviruses are thought to transmit orally via saliva. MuHV-4 may use nasal entry because noses feature more prominently in murine social life. The nasopharynx may also be important for human gammaherpesvirus transmission: there is little evidence for a specifically oral EBV or KSHV entry route, and EBV infection notably predisposes to nasopharyngeal carcinoma.

The limited lytic spread of MuHV-4 from the nose might be seen as suboptimal for host colonization. However, gammaherpesvirus epidemiology indicates that transmission depends more on long-term virus shedding than primary lytic infection, and this correlates with the latent load (Yao *et al.*, 1985). MuHV-4 latency is relatively independent of the extent of primary lytic infection (Stevenson *et al.*, 1999c; Coleman *et al.*, 2003). It depends much more on latency-associated lymphoproliferation (May *et al.*, 2004). Extensive primary lytic spread might even be counter-productive by providing a powerful immune stimulus and by predisposing the host to disease. Thus, gammaherpesviruses may have evolved to infect their hosts without extensive lytic spread.

The apparent failure of intranasal MuHV-4 to infect the oropharynx or trachea, and of oral MuHV-4 to infect anywhere, suggest that incoming virions cross epithelia by specialized routes. Although MuHV-4 virions readily infect most epithelial cells *in vitro*, their strong dependence on heparan sulphate for cell binding (Gillet *et al.*, 2008) raises questions about epithelial infection *in vivo*, as here heparan sulfate is predominantly basolateral rather than apical (Hayashi *et al.*, 1987). *In vitro* epithelial infection may correspond more to host exit, when virions would emerge from B cells to infect basolaterally. Notably, MuHV-4 infects confluent, polarized *in vitro* epithelial monolayers much less well than subconfluent monolayers (our unpublished data).

An important task now is to identify the cell types targeted in the nose. This is not necessarily straightforward: high dose inocula may reach non-physiological sites, low dose inocula are inherently hard to track, and once there is viral replication, histology may fail to distinguish host entry from exit. Entry via the nose may be qualitatively different to that via the lungs or the peritoneal cavity. The latter both contain abundant macrophage populations without an epithelial barrier, and peritoneal macrophages at least are quite readily infected (Rosa *et al.*, 2007). A related question is how incoming virions reach B cells. This may occur submucosally or in lymph nodes. The strong luciferase signals in SCLN following nasal infection argued for a significant lymphatic transport of infectious virions to this site. One precedent for such transport is normal immune priming (Belz *et al.*, 2007). A role for dendritic cells in B-cell infection would therefore not be surprising. The present study provides a basis for further understanding by identifying the nose as an entry point and the SCLN as the major associated lymphoid target.
